# Lynch Syndrome - is breast cancer a feature?

**DOI:** 10.1186/1897-4287-9-S1-P1

**Published:** 2011-03-10

**Authors:** Zohra Ali-Khan Catts, Carrie Barnum, Marcie Parker, Chandra Somerman, Becky Grey, Bruce Boman

**Affiliations:** 1 Familial Cancer Risk Assessment Program, Helen F. Graham Cancer Center, Christiana Care Health System, Newark DE 19713, USA

## Background

The debate on whether or not breast cancer is in the tumor spectrum for Lynch syndrome produces a conundrum for healthcare providers. The classic tumor spectrum for Lynch Syndrome (LS) includes colon, endometrial, ovarian, stomach, small intestine, hepatobiliary, urinary tract and brain/central nervous system cancers. Muir-Torre Syndrome (MTS) is a variant of LS that is associated with additional skin lesions including sebaceous gland tumors and keratoacanthomas. MTS was observed in 28% of LS families when assessing for MTS skin lesions [1]. It has also been reported that 10-14% of individuals with MTS present initially with breast cancer [2,3]. An extensive study published in 2002 excluded breast cancer as part of the tumor spectrum associated with LS [4]. However, more recently it was reported that DNA mismatch repair (MMR) gene deficiencies were identified in 51% of breast cancers arising in MMR mutation carriers [5]. Another study reported a male with an MLH1 mutation who had both colon and breast cancer. The breast cancer exhibited somatic reduction to homozygosity for the MLH1 mutation [6]. Here we report two unrelated families in which the proband has a germline MMR gene mutation and bilateral breast cancer, and one family in which the proband had ovarian and renal cancer and her daughter, maternal aunt and cousin had breast cancer at age 47, 59, and 48 respectively. This raises the question are these breast cancers associated with the MMR mutations or a breast cancer susceptibility gene and what testing should be offered?

## Methods

Three families underwent genetic counseling and testing through the Familial Cancer Risk Assessment Program and were found to have mutations in an MMR gene. All three families were then offered BRCA1/BRCA2 testing based on a personal and/or family history suggestive of hereditary breast and ovarian cancer. Informed consent was obtained and the proband underwent BRCA1/BRCA2 testing.

## Results

All three probands underwent BRCA1/BRCA2 DNA sequencing and one proband had large rearrangement analysis performed. All three probands were negative for alterations in BRCA1/BRCA2 genes.

## Conclusion

Our findings indicate that breast cancer is part of the spectrum of tumors in LS families in which the breast cancer segregates with the other LS associated tumors. Additional hereditary breast cancer gene testing may not be warranted in these circumstances. A future research goal is to perform IHC on the breast tumors from these families to determine if they show loss of expression of the MMR gene that is known to be altered.
